# Priorities, Barriers, and Facilitators towards International Guidelines for the Delivery of Supportive Clinical Care during an Ebola Outbreak: A Cross-Sectional Survey

**DOI:** 10.3390/v11020194

**Published:** 2019-02-23

**Authors:** Marie-Claude Battista, Christine Loignon, Lynda Benhadj, Elysee Nouvet, Srinivas Murthy, Robert Fowler, Neill K. J. Adhikari, Adnan Haj-Moustafa, Alex P. Salam, Adrienne K. Chan, Sharmistha Mishra, Francois Couturier, Catherine Hudon, Peter Horby, Richard Bedell, Michael Rekart, Jan Hajek, Francois Lamontagne

**Affiliations:** 1 Centre de recherche du CHUS de Sherbrooke, Sherbrooke, QC J1H 5N4, Canada; Marie-Claude.Battista@USherbrooke.ca (M.-C.B.); Catherine.Hudon@USherbrooke.ca (C.H.); 2 Department of Family Medicine and Emergency Medicine, Université de Sherbrooke, Sherbrooke, QC J1H-5N4, Canada; Christine.Loignon@USherbrooke.ca (C.L.); Francois.Couturier@USherbrooke.ca (F.C.); 3 Centre de recherche, Hôpital Charles-Le Moyne, Longueuil, QC J4V 2H1, Canada; lynda.benhadj@usherbrooke.ca; 4 Department of Community Health Sciences, Université de Sherbrooke, Sherbrooke, QC J1H 5H3, Canada; 5 Western University, School of Health Studies, London, ON N6A 3K7, Canada; enouvet@uwo.ca; 6 Department of Paediatrics, University of British Columbia, Vancouver, BC V6H 3N1, Canada; srinivas.murthy@cw.bc.ca; 7 Interdepartmental Division of Critical Care Medicine, University of Toronto, Toronto, ON, Canada; rob.fowler@sunnybrook.ca (R.F.); neill.adhikari@utoronto.ca (N.K.J.A.); 8 Department of Critical Care Medicine, Sunnybrook Health Sciences Centre, Toronto, ON M4N 3M5, Canada; 9 Clinical Research Unit, Research Center, Sainte-Justine Hospital, Université de Montréal, Montréal, QC H3S 2G4, Canada; adnanhm2@hotmail.com; 10 Outbreak Diseases Research Group, University of Oxford, Wellcome Trust Centre for Human Genetics, Oxford OX3 7BN, UK; alex.salam@ndm.ox.ac.uk (A.P.S.); peter.horby@ndm.ox.ac.uk (P.H.); 11 Division of Infectious Diseases, Department of Medicine, Sunnybrook Health Sciences Centre, University of Toronto, Toronto, ON M4N 3M5, Canada; adrienne.chan@sunnybrook.ca; 12 Dignitas International, Zomba P.O. Box 1071, Malawi; 13 Centre for Urban Health Solutions, Li Ka Shing Knowledge Institute, St. Michael’s Hospital, University of Toronto, Toronto, ON M5S 1J4, Canada; mishras@smh.ca; 14 Division of Global Health, University of British Columbia, Vancouver, BC V6T-1Z3, Canada; bedellrichard@gmail.com; 15 School of Population and Public Health, University of British Columbia, Vancouver, BC V6T-1Z3, Canada; Michael.rekart@bccdc.ca; 16 Department of Medicine, Division of Infectious Diseases, University of British Columbia, Vancouver, BC V6T 1Z3, Canada; janjhajek@gmail.com; 17 Department of Medicine, Division of Critical Care Medicine, Université de Sherbrooke, Sherbrooke, QC J1H 5N4, Canada

**Keywords:** survey, supportive care, priorities, barriers and facilitators, Ebola Virus Disease

## Abstract

During the Ebola outbreak, mortality reduction was attributed to multiple improvements in supportive care delivered in Ebola treatment units (ETUs). We aimed to identify high-priority supportive care measures, as well as perceived barriers and facilitators to their implementation, for patients with Ebola Virus Disease (EVD). We conducted a cross-sectional survey of key stakeholders involved in the response to the 2014–2016 West African EVD outbreak. Out of 57 email invitations, 44 responses were received, and 29 respondents completed the survey. The respondents listed insufficient numbers of health workers (23/29, 79%), improper tools for the documentation of clinical data (*n* = 22/28, 79%), insufficient material resources (*n* = 22/29, 76%), and unadapted personal protective equipment (*n* = 20/28, 71%) as the main barriers to the provision of supportive care in ETUs. Facilitators to the provision of supportive care included team camaraderie (*n* in agreement = 25/28, 89%), ability to speak the local language (22/28, 79%), and having treatment protocols in place (22/28, 79%). This survey highlights a consensus across various stakeholders involved in the response to the 2014–2016 EVD outbreak on a limited number of high-priority supportive care interventions for clinical practice guidelines. Identified barriers and facilitators further inform the application of guidelines.

## 1. Introduction

Ebola Virus Disease (EVD) is a febrile illness that often leads to gastrointestinal fluid losses and complications of hypovolemia, including renal dysfunction, metabolic acidosis, and organ dysfunction [1,2,3,4,5]. The optimal clinical care of these patients includes supportive care to prevent and manage organ dysfunction [2,3], which in turn requires adequately staffed and resourced Ebola treatment units (ETUs) with appropriate infection prevention and control protocols. During the 2014–2016 West Africa EVD outbreak, the provision of supportive care varied across ETUs and over time. While previous reports identified factors that motivated or hindered the mobilization of health professionals [6,7], there were no clinical practice guidelines on a core set of supportive care measures that should be guaranteed to all patients treated in ETUs. 

While the overall mortality rate reached 92% in the early phase of the outbreak (August–September 2014) in one country, the rate was 67% for those admitted to ETUs [8]. The observed mortality reduction has been attributed to earlier patient presentation, but also to multiple gradual improvements in supportive care delivered in ETUs as staff expertise and material resources increased [2]. Based on this observation, we launched a program of research that culminated in the publication of evidence-based guidelines for the supportive care of patients with EVD [9]. To inform the development of these guidelines, we designed and executed a cross-sectional survey of key stakeholders involved in the response to the 2014–2016 West African EVD outbreak. The specific objective of the survey was to identify a short list of high-priority candidate supportive care interventions to be incorporated in the guidelines [9]. A secondary objective was to collect information on perceived barriers and facilitators to the provision of supportive care during the outbreak. 

## 2. Materials and Methods 

### 2.1. Sampling Frame

Between January and June 2016, email invitations to complete the online survey were sent to individuals identified as having been involved in the response to the 2014–2016 West African EVD outbreak. Using a non-probabilistic, purposive sampling frame (i.e., snowball), we initially identified individuals known to the research team and subsequently relied on suggestions from the first wave of participants to recruit additional respondents across the spectrum of relevant stakeholders (i.e., decision makers, physicians, nurses, age groups, sex, country of residence, governmental versus non-governmental affiliations, different periods during the outbreak). Individuals who responded to the invitation email at least once were identified as respondents. Unfortunately, given that we often lacked contact information sampling continued until the research team felt that adequate representation had been achieved. Members of the research team were not eligible to become survey participants. Sampling was not stratified. 

### 2.2. Questionnaire Development

The survey protocol followed published recommendations for self-administered surveys of healthcare workers [10]. The research team conducted in-depth focus group sessions with content experts to identify important domains and specific issues within domains, highlighting those that were most pertinent to the provision of supportive care during an EVD outbreak. We explicitly focused on the domains of (1) the adequacy of clinical care delivered during the outbreak, (2) barriers to the provision of supportive care, (3) facilitators to the provision of supportive care, (4) potential solutions to improve care, and (5) benchmark measures of minimally acceptable supportive care. We categorized barriers, facilitators, and potential solutions as issues pertaining to supplies and technology, personnel, and organizational structure. Demographic information was entered as free text, but respondents answered all other questionnaire items using seven-category Likert scales. 

### 2.3. Questionnaire Testing

Three members of the research team who were involved as health professionals during the 2014–2016 West African EVD outbreak tested the first version of the questionnaire and provided feedback regarding its clarity and ease of administration. Following an iterative process of item generation and reduction, we then assessed the questionnaire’s clinical sensibility (i.e., whether the questionnaire specifically addressed the five domains) by administering the questionnaire to six content experts who had not been involved in its design. We revised the questionnaire based on the feedback provided. A bilingual member of the research team who was involved as a health professional during the 2014–2016 West African EVD outbreak drafted the first version of the questionnaire and translated the final version from English to French to ensure the consistency of both final versions.

### 2.4. Questionnaire Administration

We administered the questionnaire using web-based software (LimeSurvey, Hamburg, Germany). Once they had provided informed consent, each participant received a link to the survey via email. The English and French versions of the questionnaire are available in Supplementary 1 and 2. We sent up to two reminders by email to participants who had provided informed consent but who did not complete the questionnaire. 

### 2.5. Statistical Analyses

Responses were summarized using proportions for categorical data and medians (interquartile ranges [IQR]) for continuous data. “Strongly disagree”, “disagree”, and “somewhat disagree” were collapsed to “disagree”; meanwhile, “strongly agree”, “agree”, and "somewhat agree" were collapsed to “agree”. “Neutral” was set as a distinct category. Associations between demographic characteristics and responses and stratification by age, gender, and affiliation type could not be analyzed due to the insufficient sample size.

### 2.6. Funding and Ethical Approval

The Population and Public Health Institute of the Canadian Institutes of Health Research (CIHR) funded this project (ER2-143490). The CIUSSS-Estrie CHUS Research Ethics Board approved the study (#2016-1257, approved 3 Feb 2016). The CIHR had no influence on the design, conduct, analysis, preparation of the manuscript, or decision to submit the manuscript for publication.

### 2.7. Patient Involvement

Patients were not involved in either the study design or dissemination of results.

## 3. Results

### 3.1. Respondent Characteristics

**Figure 1:**
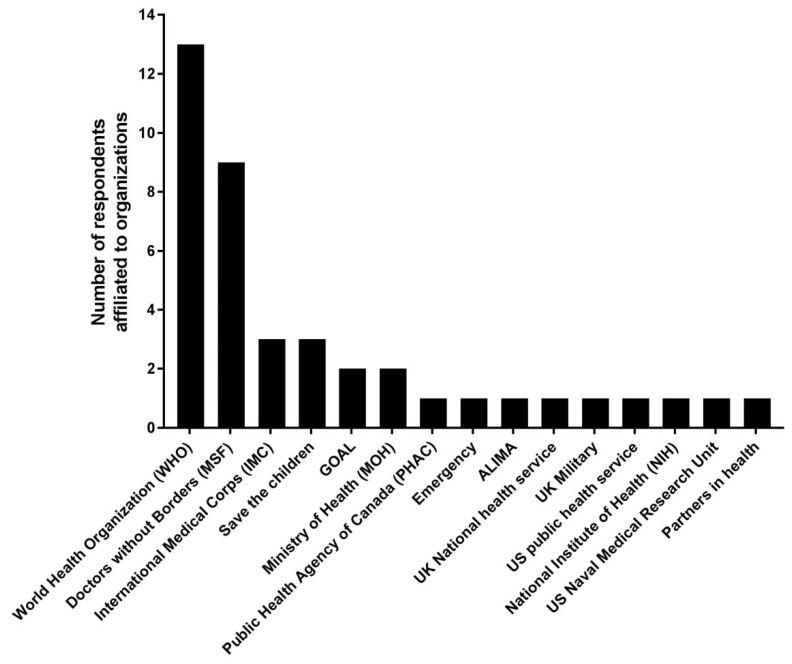
Professional affiliations reported by survey respondents.

**Figure 2:**
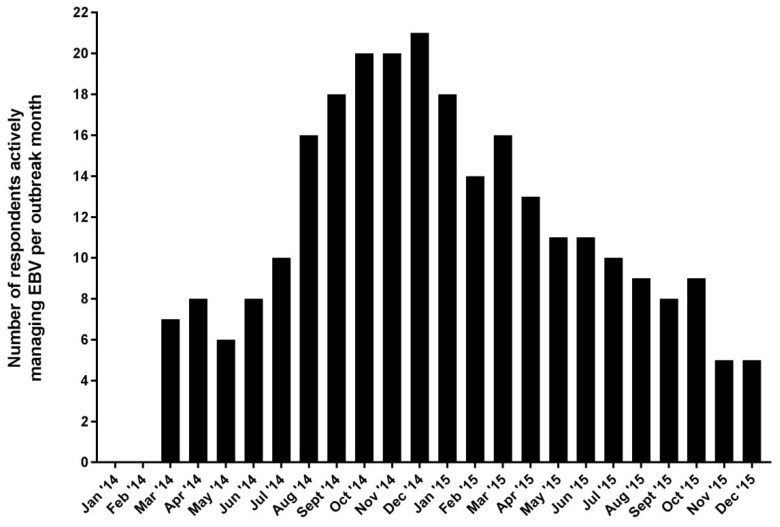
Number of active respondents by outbreak period.

Between January and June of 2016, 57 individuals received an invitation to participate in the survey, and 44 replied to at least one email for a response rate of 77%. Of these, 29 agreed to participate, for a participation rate of 66%. Nine provided consent, but did not complete the questionnaire, and 19 either declined the invitation or did not respond to at least one email invitation. Demographic characteristics are summarized in Table 1. Twenty-five respondents (86%) completed the survey in English, and four (14%) completed the survey in French. The median age of respondents was 40 (IQR 34, 48), eight (28%) were women, and 11 (38%) resided in an African country, five of whom (17%) were citizens of Guinea (*n* = 2), Liberia (*n* = 1), or Sierra Leone (*n* = 3). Respondents indicated up to three professional affiliations (Figure 1), but the survey responses reflect the participants’ personal views, rather than the views of their organizations. Twenty-four respondents (83%) were physicians, three were nurses (10%), and two were responsible for project management and coordination (7%). Between March 2014 and December 2015, the 23 respondents who provided clinical care treated patients in a median of three different ETUs (IQR 2, 4) for a median of 10 weeks total (IQR 8, 20). One participant who identified as a project manager declined to answer questions pertaining to the provision of clinical care, which he considered outside his area of expertise. Figure 2 illustrates at which periods respondents were involved in the outbreak response. 

### 3.2. Barriers, Facilitators, and Supportive Care Priorities

Most respondents (*n* = 27/29; 93%) believed that they were qualified to assess the quality of care delivered in ETUs and disagreed with the statement that care was optimal (*n* = 27/29; 93%). Respondents believed that the barriers with the largest impact on the provision of supportive care were (in decreasing order of importance) insufficient numbers of healthcare workers (maintenance, surveillance, laboratory professionals; *n* = 23/29, 79%), improper tools for the documentation of clinical data (*n* = 22/28, 79%), insufficient numbers of physicians and nurses (*n* = 22/29, 76%), insufficient material resources (drug supplies, intravenous catheters and lines; *n* = 22/29, 76%), suboptimal personal protective equipment (*n* = 20/28, 71%), limited communication between organizations (limited sharing of protocols, advice, standards of care, endorsements for intensified therapy, *n* = 17/28, 61%), poorly defined roles and responsibilities (*n* = 16/29, 55%), pressure to care for non-EVD patients in the context of a failing healthcare system (*n* = 13/28, 46%), and limited communication within organizations (limited sharing of protocols, advice, standards of care, and endorsements for intensified therapy, *n* = 12/29, 41%). 

In contrast, team camaraderie (*n* = 25/28, 89%), fluency in the Ebola-affected country’s official languages (*n* = 22/28, 79%), treatment protocols in place (*n* = 22/28, 79%), acute critical care expertise (*n* = 20/28, 71%), examples of treatment successes (*n* = 20/28, 71%), clinician autonomy (*n* = 15/28, 54%), and expertise in infectious diseases (*n* = 14/28, 50%) were perceived as facilitators. 

**Figure 3:**
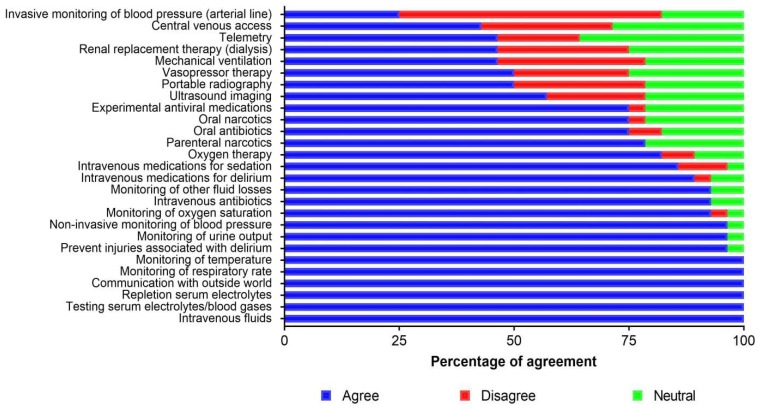
Agreement with supportive care interventions proposed to be embedded within standard practices in Ebola treatment units.

Figure 3 illustrates to what extent respondents believed individual interventions listed in the survey tool should become standards of ETU care. No respondent disagreed with statements proposing that the following 12 interventions should be systematically available to all patients treated in ETUs: (1) intravenous fluids (*n* agree = 28), (2) testing of serum biochemistry (*n* agree = 28), (3) correction of biochemical anomalies (*n* agree = 28), (4) methods to enable communication with relatives and friends (*n* agree = 28), (5) non-invasive monitoring of respiratory rate (*n* agree = 28); (6) temperature (*n* agree = 28) and (7) blood pressure (*n* agree = 27; *n* neutral = 1), (8) monitoring of urine output (*n* = 27), (9) early recognition of clinical deterioration and the prevention of injuries (*n* agree = 27; *n* neutral = 1), (10) intravenous antibiotics (*n* agree = 26; *n* neutral = 2), (11) monitoring of gastrointestinal fluid losses (*n* agree = 26; *n* neutral = 2), and (12) intravenous opioids for treatment of pain (*n* agree = 22; *n* neutral = 6).

### 3.3. Auditing the Delivery of Care in ETUs

When asked whether organizations (governmental, intergovernmental, or non-governmental) monitored the quality of care in ETUs, 13/28 (46%) respondents agreed, 2/28 (7%) were neutral, and 13/28 (46%) disagreed. Out of 28 respondents, 14 (50%) disagreed that health workers in ETUs knew which interventions constituted benchmarks for high-quality care, 5/28 (18%) were neutral, and 9/28 (29%) agreed. 

## 4. Discussion

This self-administered survey of 29 health workers involved at different levels and periods during the response to the 2014–2016 West African EVD outbreak informed the development of evidence-based guidelines for the supportive care of patients treated in ETUs. [9] A short list of high-priority interventions was created and used during subsequent phases of the guidelines’ development process. Moreover, the identification of perceived barriers and facilitators affecting the provision of supportive care in ETUs constitute valuable information relevant to the application of the guidelines. 

The results of this survey echo various reports describing health professionals’ perceptions of the supportive care that was delivered in ETUs during the outbreak. As more individuals advocated for better supportive care measures, the provision of interventions that were perceived as high priorities by survey respondents also increased [2,11,12]. A qualitative analysis of semi-structured interviews conducted with each survey respondent provides further insight into the barriers and facilitators elicited by this survey [13]. For example, the fluctuating case load, perceived inhumanity of patients’ suffering separate from their kin, variable diagnostic and treatment resources available at different points in the epidemic and in different ETUs, and variable clinical experience, are part of the many nuances that might help understand the challenges identified in the survey [13].

This survey met accepted methodologic standards [10], used purposive sampling to ensure at least the minimal representation of key relevant stakeholder groups, and the results informed clinical practice guidelines published in a high-impact journal [9]. However, we acknowledge a number of limitations. The sample size was small, which limits the generalizability of the results. We attribute the small sample size to the challenges associated with identifying potential respondents using very limited contact information and the constrained means of communicating during the outbreak. Relying on different modes of communication that are less vulnerable to electricity shortages or low-bandwidth internet access (e.g., WhatsApp) may have improved the efficiency of the research design, but this would have required access to personal telephone numbers which, in contrast to email addresses, are not typically found in the public domain. This survey’s sample size is comparable to a similar survey that was completed by 44 frontline physicians and nurses, which led to the elaboration of World Health Organization (WHO) recommendations on personal protective equipment [14]. Another potential limitation is that responses were obtained mostly from World Health Organization and Doctors Without Borders (MSF) members, with only a minority from regional authorities and the Ministry of Health. The latter will have a better understanding of local healthcare issues (e.g., healthcare practices, communication strategies) and baseline resource limitations in the affected countries. As such, the identified barriers may be more reflective of perceptions of the international health and humanitarian community rather than the local healthcare community, and thus concrete measures in response to these barriers would benefit from further input from local healthcare teams. We assumed that minimal knowledge of clinical management was a mandatory requirement to discuss the provision of supportive care in ETUs. However, ultimately, the guideline panel was allowed to build upon the list of high-priority interventions identified during the survey exercise [9], grouping certain interventions in a single recommendation, or adding more interventions as relevant. The small sample size prevented secondary analyses that might have shed light on the factors associated with specific responses (e.g., respondent gender, affiliation, geography, or speciality/expertise). However, the focus of this survey was not to explain variations in opinions between different stakeholders, but rather to identify interventions that were unanimously perceived as high priorities by all stakeholders. There was no variation in the responses pertaining to the interventions that were ultimately short-listed and shared with the guideline panel. Admittedly, quantitative surveys do not provide in-depth knowledge of the respondents’ views on complex issues, which are better captured by qualitative research designs. As mentioned, understanding that a qualitative analysis would enhance future guideline panel discussions, we also conducted a complementary qualitative study [13].

## 5. Conclusions

In this survey of health professionals involved in the international response to the 2014–2016 EVD outbreak, there was consensus on several supportive care interventions that should become standard practice across all ETUs and could become benchmarks for quality of care in the future.

## Figures and Tables

**Table 1:** Respondents’ demographic characteristics.

Characteristics			Respondents (*n* = 29)	^1^Provided consent, but did not completed questionnaire (*n* = 9)	^1^Declined invitation or did not respond to invitation (*n* = 19)
**Age–median [IQR]**			40 [34, 48]	Footnote ^4^	
**Sex–n female (%)**			8 (28)	1 (11)	7 (37)
**English–*n* (%)**			25 (86)	Footnote^4^	
**Residents of African countries–*n* (%)**	All countries		11 (38)	Footnote^4^	
	Guinea, Liberia or Sierra Leone		5 (17)		
**Expertise–*n* (%)**	**Medicine ^2^**	Public health & epidemiology	5 (17)	6 (67)	16 (84)
		Infectious diseases	4 (14)		
		Other (anaesthesiology, emergency medicine, pediatrics)	3 (10)		
		Unspecified	14 (48)		
	**Nursing**		3 (10)	1 (11)	2 (11)
	**Project management—coordination**		3 (10)	2 (22)	1 (5)
**Years of experience–** **median [IQR]**			13 [6, 17]	Footnote^4^	
**^3^Country of usual professional activities–*n* (%)**	United States of America		9 (31)		
	Sierra Leone		8 (28)		
	Guinea		5 (17)		
	United Kingdom		5 (17)		
	Uganda		4 (14)		
	Switzerland		3 (10)		
	Canada		3 (10)		
	Other (Peru, Kenya, Malawi, India, Haiti, Honduras, Ethiopia, South Sudan, Croatia, Sudan, Italy, Afghanistan, Turkey, Ireland, Australia, Belgium, Liberia, France, Senegal, Mali, Niger, Democratic Republic of Congo, Chad, Burkina Faso, Cameroon)		8 (28)		
**Number of affiliations–*n* (%)**	Single affiliation		19 (66)	Footnote^4^	
	Two affiliations		8 (28)		
	Three affiliations		2 (7)		
**Main affiliation type**	Governmental		5 (17)	3 (33)	6 (32)
	Non-governmental		24 (83)	6 (66)	13 (68)

^1^ Demographic characteristics could not be verified and should be interpreted with caution. ^2^ Respondents could enter more than one expertise. ^3^ Respondents could enter more than one country. ^4^ Sharing of this information was not authorized. IQR: Interquartile range.

## Supplementary Materials

The following are available online at https://www.mdpi.com/1999-4915/11/2/194/s1, Supplementary 1: Priorities, barriers and facilitators towards international guidelines for the delivery of supportive clinical care during an Ebola outbreak—a cross-sectional survey (English version of the survey); Supplementary 2: Prestation de soins de soutien au cours d’une éclosion d’Ebola: définir les exigences minimales et identifier les obstacles et les mesures facilitantes dans le but d’élaborer des lignes directrices internationals (French version of the survey).
