# Supplementary material for: Priorities, Barriers, and Facilitators towards International Guidelines for the Delivery of Supportive Clinical Care during an Ebola Outbreak: A Cross-Sectional Survey
Source: Viruses. 2019 Feb 23;11(2):194. doi: 10.3390/v11020194 (PMC6409845; doi:10.3390/v11020194)
Supplement: Supplementary file 1 [file viruses-11-00194-s001.zip › Supplementary 1_-_Ebola_Quantitative_Survey_ENGLISH.docx]

**Interview with decision-makers and clinicians**

**Quantitative Survey**

**Demographics:**

1. Age:
2. Country of usual professional activities (if multiple list all):
3. Title (nurse, physician, logistician, administrator, etc.):
   - Specialty :
4. Number of years in practice:
5. For clinicians, for how many weeks have you been involved in the delivery of care to patients during the Ebola outbreak? ENTER NUMBER; in how many different Ebola treatment units? ENTER NUMBER
6. During the most recent 2014-15 Ebola viral disease outbreak, what organization(s) did you work for?:
7. During the most recent 2014-15 Ebola viral disease outbreak, what was your role: FREE TEXT THAT WE WILL CATEGORIZE LATER
8. During which months were you actively involved in the response to the recent Ebola outbreak: CHECK BOXES FOR EACH MONTH OF INVOLVEMENT
9. During your involvement in the recent Ebola outbreak, where were you based (all that apply)? Please specify country and name of facility.

**To what extent do you agree with this series of statements? For each section, please consider that 'delivery of care' refers to all aspects of clinical management within Ebola treatment units in West Africa, whether patients had a confirmed infection or were simply being monitored.**

|  | Strongly Disagree | Disagree | Somewhat Disagree | Neutral | Somewhat Agree | Agree | Strongly Agree | |
| --- | --- | --- | --- | --- | --- | --- | --- | --- |
| 1. Your assessment of care delivered within Ebola treatment units (ETUs) | | | | | | | |  |
| - 1. My professional background enables me to comment on the clinical management of patients infected with Ebola. |  |  |  |  |  |  |  | |
| - 1. My involvement during the Ebola viral disease outbreak led me to think about the delivery of care within ETUs even before I was invited to participate to this survey |  |  |  |  |  |  |  | |
| 1. Perception of barriers that limited the delivery of clinical care in ETUs | | | | | | | |  |
| - 1. There were no barriers to care and clinical management was optimal (i.e. patients would have received the same care anywhere in very developed countries) |  |  |  |  |  |  |  | |
| - 1. Insufficient material resources (i.e. drug supplies, intravenous catheters and lines, etc.) limited the delivery of care |  |  |  |  |  |  |  | |
| - 1. An insufficient number of physicians limited the delivery of care |  |  |  |  |  |  |  | |
| - 1. An insufficient number of nurses limited the delivery of care |  |  |  |  |  |  |  | |
| - 1. An insufficient number of health workers (e.g. maintenance, surveillance, laboratory professionals, etc.) limited the delivery of care |  |  |  |  |  |  |  | |
| - 1. Poorly defined roles and responsibilities limited the delivery of care |  |  |  |  |  |  |  | |
| - 1. Communication within your organization limited the delivery of care (limited sharing of protocols, advice, standards of care, endorsements for intensified therapy, etc.) |  |  |  |  |  |  |  | |
| - 1. Communication between your and other organizations limited the delivery of care (limited sharing of protocols, advice, standards of care, endorsements for intensified therapy, etc.) |  |  |  |  |  |  |  | |
| - 1. Improper tools for the documentation of clinical data (e.g. medical records) limited the delivery of care |  |  |  |  |  |  |  | |
| - 1. Pressure to care for non-EVD patients in the context of a failing healthcare system limited the delivery of care |  |  |  |  |  |  |  | |
| - 1. Unadapted personal protective equipment limited the delivery of care |  |  |  |  |  |  |  | |
| 1. Your assessment of facilitators that aided the delivery of clinical care in ETUs | | | | | | | | |
| - 1. Team camaraderie facilitated the delivery of care |  |  |  |  |  |  |  | |
| - 1. Examples of favorable responses to therapy facilitated the delivery of care |  |  |  |  |  |  |  | |
| - 1. The treatment protocols in place facilitated the delivery of care |  |  |  |  |  |  |  | |
| - 1. Clinician autonomy facilitated the delivery of care |  |  |  |  |  |  |  | |
| - 1. The team's infectious disease expertise facilitated the delivery of care |  |  |  |  |  |  |  | |
| - 1. The team's expertise in the care of critically ill patients (e.g. intensive care, emergency medicine, pediatric resuscitation, etc.) facilitated the delivery of care |  |  |  |  |  |  |  | |
| - 1. Being able to speak the same language as the national health workers facilitated the delivery of care |  |  |  |  |  |  |  | |
| 1. Your assessment of the quality of care monitoring measures | | | | | | | | |
| - 1. Active monitoring of the quality of care (audits) took place during my missions |  |  |  |  |  |  |  | |
| - 1. Individuals working within ETUs knew what benchmark measures were used to assess quality of care |  |  |  |  |  |  |  | |
| 1. Please state to what extent you agree that the following interventions constitute minimal standards and should be made available to patients infected by the Ebola virus | | | | | | | | |
| 5.1 Intravenous fluids |  |  |  |  |  |  |  | |
| 5.2 Testing of serum electrolytes and blood gases |  |  |  |  |  |  |  | |
| 5.3 Repletion of serum electrolytes |  |  |  |  |  |  |  | |
| 5.4 Intravenous antibiotics |  |  |  |  |  |  |  | |
| 5.5 Oral antibiotics |  |  |  |  |  |  |  | |
| 5.6 Parenteral narcotics |  |  |  |  |  |  |  | |
| 5.7 Oral narcotics |  |  |  |  |  |  |  | |
| 5.8 Intravenous medications for sedation |  |  |  |  |  |  |  | |
| 5.9 Intravenous medications for delirium |  |  |  |  |  |  |  | |
| 5.10 Monitoring of urine output |  |  |  |  |  |  |  | |
| 5.11 Monitoring of other fluid losses |  |  |  |  |  |  |  | |
| 5.12 Ability to communicate with outside world/family |  |  |  |  |  |  |  | |
| 5.13 Constant monitoring and ability to prevent injuries associated with delirium |  |  |  |  |  |  |  | |
| 5.14 Oxygen therapy |  |  |  |  |  |  |  | |
| 5.15 Mechanical ventilation |  |  |  |  |  |  |  | |
| 5.16 Renal replacement therapy (dialysis) |  |  |  |  |  |  |  | |
| 5.17 Vasopressor therapy |  |  |  |  |  |  |  | |
| 5.18 Non-invasive onitoring of blood pressure |  |  |  |  |  |  |  | |
| 5.19 Invasive monitoring of blood pressure (arterial line) |  |  |  |  |  |  |  | |
| 5.20 Central venous access |  |  |  |  |  |  |  | |
| 5.21 Telemetry |  |  |  |  |  |  |  | |
| 5.22 Monitoring of oxygen saturation |  |  |  |  |  |  |  | |
| 5.23 Monitoring of respiratory rate |  |  |  |  |  |  |  | |
| 5.24 Monitoring of temperature |  |  |  |  |  |  |  | |
| 5.25 Portable radiography |  |  |  |  |  |  |  | |
| 5.26 Ultrasound imaging |  |  |  |  |  |  |  | |
| 5.27 Experimental antiviral medications |  |  |  |  |  |  |  | |
| 5.28 Other (enter free text): |  | | | | | | | |
|  |  | | | | | | | |
|  |  | | | | | | | |
|  |  | | | | | | | |
|  |  | | | | | | | |
|  |  | | | | | | | |
|  |  | | | | | | | |
|  |  | | | | | | | |
|  |  | | | | | | | |
|  |  | | | | | | | |
|  |  | | | | | | | |
