# Supplementary material for: Priorities, Barriers, and Facilitators towards International Guidelines for the Delivery of Supportive Clinical Care during an Ebola Outbreak: A Cross-Sectional Survey
Source: Viruses. 2019 Feb 23;11(2):194. doi: 10.3390/v11020194 (PMC6409845; doi:10.3390/v11020194)
Supplement: Supplementary file 1 [file viruses-11-00194-s001.zip › Supplementary 2_Ebola_Quantitative_Survey_FRENCH.docx]

**Entrevue auprès des décideurs et des cliniciens**

**Enquête quantitative**

**Données démographiques :**

1. Âge :
2. Pays où vous exercez normalement vos activités professionnelles (si plusieurs, veuillez préciser tous les pays) :
3. Titre (infirmière, médecin, logisticien, administrateur, etc.) :
   - Spécialité :
4. Nombre d’années d’exercice :
5. À l’intention des cliniciens : pendant combien de semaines avez-vous été impliqué(e) dans la prestation des soins de santé aux patients au cours de cette éclosion d’Ebola? VEUILLEZ ENTRER LE NOMBRE DE SEMAINES ; dans combien d’unités de traitement d’Ebola (UTE) distinctes avez-vous travaillé (si aucune, inscrivez 0)? VEUILLEZ ENTRER LE NOMBRE D’UTE.
6. Lors de l’éclosion de la maladie à virus Ebola (MVE) la plus récente en 2014-2015, avec quelle(s) organisation(s) avez-vous travaillé ?
7. Lors de l’éclosion de la MVE la plus récente en 2014-2015, quel a été votre rôle ? TEXTE LIBRE À CATÉGORISER ULTÉRIEUREMENT
8. Pendant quels mois avez-vous été activement impliqué dans la réponse à l’éclosion de la MVE récente ? VEUILLEZ COCHER TOUTES LES CASES CORRESPONDANTES À CHAQUE MOIS PENDANT LEQUEL VOUS AVEZ PARTICIPÉ À CETTE INTERVENTION CONTRE L’ÉCLOSION DE LA MVE.
9. Lors de votre participation à l’intervention menée contre l’éclosion de la MVE récente, où étiez-vous affecté (tous les sites qui s’appliquent) ? Veuillez préciser le nom du ou des pays ainsi que le nom de l’ ou des installations.

**Veuillez indiquer dans quelle mesure vous êtes en accord avec cette série d’énoncés. Pour chaque section, veuillez noter que par « prestation de soins », on entend tous les aspects de la prise en charge clinique des patients dans les UTE situées en Afrique de l’Ouest, qu’il s’agisse de patients ayant une infection confirmée à Ebola ou de patients sous surveillance (cas suspects) simplement.**

|  | Tout-à-fait en désaccord | En désaccord | Plutôt en désaccord | Ni en accord ni en désaccord | Plutôt en accord | En accord | Tout-à-fait en accord | |
| --- | --- | --- | --- | --- | --- | --- | --- | --- |
| 1. Votre évaluation des soins dispensés dans les UTE | | | | | | | |  |
| - 1. Mon bagage professionnel facilite ma réflexion sur la prise en charge des patients infectés par le virus Ebola. |  |  |  |  |  |  |  | |
| - 1. Mon implication lors de l’éclosion de la MVE m’a amené à réfléchir sur la prestation des soins dans les UTE même avant d’être invité(e) à participer à la présente enquête. |  |  |  |  |  |  |  | |
| 1. Perception des obstacles à la prestation des soins cliniques dans les UTE | | | | | | | |  |
| - 1. Il n’y avait pas d’obstacle à la prestation des soins. La prise en charge clinique était optimale (les patients auraient reçu les mêmes soins n’importe où dans les pays très développés). |  |  |  |  |  |  |  | |
| - 1. Les ressources matérielles insuffisantes (approvisionnement en médicaments, cathéters et tubes intraveineux, etc.) ont nui à la prestation des soins. |  |  |  |  |  |  |  | |
| - 1. Le nombre insuffisant de médecins a nui à la prestation des soins. |  |  |  |  |  |  |  | |
| - 1. Le nombre insuffisant d’infirmières a nui à la prestation des soins. |  |  |  |  |  |  |  | |
| - 1. Le nombre insuffisant de travailleurs de la santé (employés de soutien, de surveillance, de laboratoire, etc.) a nui à la prestation des soins. |  |  |  |  |  |  |  | |
| - 1. Les fonctions et rôles mal définis ont nui à la prestation des soins. |  |  |  |  |  |  |  | |
| - 1. La communication au sein de mon organisation a nui à la prestation des soins (faible partage de protocoles, de conseils, de normes de soins, d’approbations pour des traitements intensifiés, etc.). |  |  |  |  |  |  |  | |
| - 1. La communication entre mon organisation et les autres organismes a nui à la prestation des soins (faible partage de protocoles, de conseils, de normes de soins, d’approbations pour des traitements intensifiés, etc.). |  |  |  |  |  |  |  | |
| - 1. L’utilisation d’outils peu appropriés pour documenter les données cliniques (ex. : dossiers médicaux) a nui à la prestation des soins. |  |  |  |  |  |  |  | |
| - 1. La pression de prendre soin des patients non atteints de la MVE dans le cadre d’un système de santé en échec a nui à la prestation des soins. |  |  |  |  |  |  |  | |
| - 1. Les équipements de protection individuelle peu adaptés ont nui à la prestation des soins. |  |  |  |  |  |  |  | |
| 1. Votre évaluation des mesures facilitantes ayant aidé à la prestation des soins cliniques dans les UTE | | | | | | | | |
| - 1. La camaraderie d’équipe a facilité la prestation des soins. |  |  |  |  |  |  |  | |
| - 1. Les exemples de réponses favorables à la thérapie ont facilité la prestation des soins. |  |  |  |  |  |  |  | |
| - 1. Les protocoles de traitement en vigueur ont facilité la prestation des soins. |  |  |  |  |  |  |  | |
| - 1. L’autonomie des cliniciens a facilité la prestation des soins |  |  |  |  |  |  |  | |
| - 1. L’expertise de l’équipe en matière de maladies infectieuses a facilité la prestation des soins |  |  |  |  |  |  |  | |
| - 1. L’expertise de l’équipe en matière de soins aux patients en phase critique (ex. : soins intensifs, médecine d’urgence, réanimation pédiatrique, etc.) a facilité la prestation des soins. |  |  |  |  |  |  |  | |
| - 1. Le fait de pouvoir parler la même langue que celle des travailleurs de la santé du pays concerné a facilité la prestation des soins |  |  |  |  |  |  |  | |
| 1. Votre évaluation des mesures visant à surveiller la qualité des soins | | | | | | | | |
| - 1. Une surveillance active de la qualité des soins (vérifications) a été menée au cours de mes missions. |  |  |  |  |  |  |  | |
| - 1. Les individus œuvrant au sein des UTE étaient au courant des points de référence servant à évaluer la qualité des soins |  |  |  |  |  |  |  | |
| 1. Veuillez indiquer à quel point vous êtes en accord avec l’énoncé que les interventions ci-dessous font partie des exigences minimales et qu’elles devraient être mises en place pour les patients infectés par le virus Ebola. | | | | | | | | |
| 5.1 Liquides intraveineux |  |  |  |  |  |  |  | |
| 5.2 Analyses des électrolytes sériques et des gaz sanguins |  |  |  |  |  |  |  | |
| 5.3 Réplétion des électrolytes sériques |  |  |  |  |  |  |  | |
| 5.4 Antibiotiques intraveineux |  |  |  |  |  |  |  | |
| 5.5 Antibiotiques oraux |  |  |  |  |  |  |  | |
| 5.6 Narcotiques parentéraux |  |  |  |  |  |  |  | |
| 5.7 Narcotiques oraux |  |  |  |  |  |  |  | |
| 5.8 Sédatifs intraveineux |  |  |  |  |  |  |  | |
| 5.9 Médicaments intraveineux pour le traitement du délire |  |  |  |  |  |  |  | |
| 5.10 Surveillance du débit urinaire |  |  |  |  |  |  |  | |
| 5.11 Surveillance d’autres pertes de liquides |  |  |  |  |  |  |  | |
| 5.12 Capacité de communiquer avec le monde extérieur ou avec la famille |  |  |  |  |  |  |  | |
| 5.13 Surveillance constante et capacité de prévenir des blessures associées au délire |  |  |  |  |  |  |  | |
| 5.14 Oxygénothérapie |  |  |  |  |  |  |  | |
| 5.15 Ventilation mécanique |  |  |  |  |  |  |  | |
| 5.16 Thérapie de remplacement rénal (dialyse) |  |  |  |  |  |  |  | |
| 5.17 Traitement vasopresseur |  |  |  |  |  |  |  | |
| 5.18 Surveillance non invasive de la tension artérielle |  |  |  |  |  |  |  | |
| 5.19 Surveillance invasive de la tension artérielle (par voie artérielle) |  |  |  |  |  |  |  | |
| 5.20 Accès veineux central |  |  |  |  |  |  |  | |
| 5.21 Télémétrie |  |  |  |  |  |  |  | |
| 5.22 Surveillance de la saturation en oxygène |  |  |  |  |  |  |  | |
| 5.23 Surveillance de la fréquence respiratoire |  |  |  |  |  |  |  | |
| 5.24 Surveillance de la température |  |  |  |  |  |  |  | |
| 5.25 Radiographie mobile |  |  |  |  |  |  |  | |
| 5.26 Imagerie à ultrasons |  |  |  |  |  |  |  | |
| 5.27 Médicaments antiviraux expérimentaux |  |  |  |  |  |  |  | |
| 5.28 Autres (insérer du texte libre) : |  | | | | | | | |
|  |  | | | | | | | |
